# Ejercer sin diploma, batallar por la licencia: encrucijadas en la historia de la profesionalización de la odontología en Colombia

**DOI:** 10.1590/S0104-59702023000100055

**Published:** 2023-10-23

**Authors:** Jorge Márquez-Valderrama, Victoria Estrada-Orrego

**Affiliations:** i Grupo de Investigación de Producción, Circulación y Apropiacón de Saberes Universidad Nacional de Colombia Medellín Antioquia Colombia jmarquez@unal.edu.co Profesor titular y coordinador del Grupo de Investigación de Producción, Circulación y Apropiacón de Saberes/ Universidad Nacional de Colombia. Medellín – Antioquia – Colombia jmarquez@unal.edu.co; ii Facultad de Artes y Humanidades Instituto Tecnológico Metropolitano Medellín Antioquia Colombia victoriaestrada@itm.edu.co Docente ocasional, Facultad de Artes y Humanidades/Instituto Tecnológico Metropolitano.Medellín – Antioquia – Colombia victoriaestrada@itm.edu.co

**Keywords:** Aprendiz, Profesionalización, Odontología, Colombia, Siglo XX, Apprenticeship, Professionalization, Dentistry, Colombia, Twentieth century

## Abstract

Este artículo estudia la profesionalización de la odontología en Colombia en la primera mitad del siglo XX. Esta historia no puede comprenderse en todas sus dimensiones si se dejan de lado las tensiones entre los practicantes con diploma y los sin diploma. Como resultado de estas tensiones, los odontólogos ganaron autonomía profesional. Analizamos solicitudes de licencia para ejercer sin título profesional, entre ellas las de algunas mujeres. Los hallazgos muestran la transmisión de conocimientos por fuera de la enseñanza formal, el ejercicio sin título y sin restricciones por parte de un gran número de odontólogos “permitidos” que se enfrentaron a una pesada y centralizada burocracia diplomada.

## Abstract (en)

This paper addresses the professionalization of dentistry in Colombia during the first half of the twentieth century. To fully comprehend such a process, we must consider the tensions between the practice of non-certified and certified dentistry. As an outcome of such tensions, dentists began to acquire professional autonomy. We analyze applications for license files to practice dentistry without a degree, some of which were of women. The findings show the informal transfer of knowledge outside formal apprenticeship and the unrestricted practice of dentistry by many non-professionals but “permitted” dentists who faced a centralized and powerful professional bureaucracy.

En las últimas cuatro décadas el estudio de las condiciones de emergencia, desarrollo y consolidación de las profesiones de la salud en América Latina se ha venido configurando como campo de investigación histórica. Los trabajos son diversos según los objetos, las orientaciones teóricas, los corpus documentales, los archivos conservados y las realidades de cada país.

Parte de los estudios se ha centrado en la profesionalización de la medicina. Algunos enfocan el problema desde un punto de vista “iatrocéntrico”, pues elaboran narrativas centradas en el acontecer de las facultades de medicina (Quevedo et al., 2010). Otros se enfocan en las prácticas concurrentes no oficiales y no diplomadas, sin contar con que la medicina diplomada tiene también su historia de contornos sociales y culturales delimitables como los de otras medicinas (Allevi, Carbonetti, 2021). También hay trabajos que analizan el proceso de constitución de la autonomía profesional y del monopolio de la actividad en contextos concretos como México y Argentina (Agostoni, 1999; Belmartino, 2010; Carrillo, 1998). Por último, están los trabajos que contextualizan el problema de la historia de los oficios de la salud desde la óptica del “pluralismo médico”, según la cual la medicina universitaria es una entre otras, no se la debería situar en el centro de una narrativa, pero tampoco se la puede ignorar cuando se estudian las llamadas “medicinas alternativas” (Armus, 2006; Armus, Gómez, 2021; Estrada-Orrego, Márquez-Valderrama, 2019).

Los procesos de profesionalización de la medicina presentan singularidades según los diferentes países; sin embargo, en ninguno la profesionalización médica aparece separada de la de oficios afines como la homeopatía, la enfermería, la farmacia, la partería y la odontología. Si bien investigaciones recientes se han interesado en abordar oficios como la partería, la enfermería y su progresiva feminización (Correa, Zárate, 2017; Martin, 2018; Ramacciotti, 2019; Zárate, 2007), en general las investigaciones sobre la historia de la profesionalización de la odontología son escasas en América Latina.

Las investigaciones históricas sobre el caso de Argentina muestran el origen artesanal y mercantil de la odontología ligado a un variado mercado de servicios, hasta alcanzar su incorporación entre los “ramos menores” de la medicina e incluso llegar a configurarse como profesión autónoma (Schapira, 2003a, 2003b). Para el caso de Brasil, hay trabajos que examinan las disputas por la instauración del monopolio de la profesión odontológica antes y después de la conformación de las escuelas de odontología (Carvalho, 2006; Lima, Nascimento, Alves, 2016; Warmling, Marzola, Botazzo, 2012). Cabe destacar el estudio consagrado a la formación y titulación de odontólogas en las primeras décadas de la República (Mott et al., 2008).

Para el caso de Colombia, hay narrativas históricas realizadas por odontólogos que se han enfocado en el problema de la institucionalización de la enseñanza formal en Bogotá y Medellín (Arango Botero, 1991; Otálora Cascante, 2017). En ese mismo conjunto hay trabajos que señalan los procesos de transmisión del oficio antes y después del establecimiento de estudios formales en el país (Duque, López, 2002; Echeverri, 1952). Sin embargo, el problema del ejercicio por parte de “odontólogos permitidos”, sin diploma universitario, pero con licencias otorgadas por organismos estatales no había sido explorado. Tampoco existen estudios sobre la participación de las mujeres en este oficio, y mucho menos sobre su presencia en el grupo de los oficiantes sin diploma.

A partir de la sociología y la historia de las profesiones se pueden reconocer varios aspectos en común en los estatutos respectivos de los oficios de médico y de odontólogo: ambos son artes del cuidado del otro; han tenido un devenir hacia “profesiones liberales”; sus practicantes han debido luchar por el prestigio y por mantener una clientela; para ambos se ha señalado la transmisión a través del modelo del aprendiz *apprenticeship* (Pelling, 2017). A pesar de esas similitudes, las diferencias entre ambos oficios, específicamente en Colombia, muestran dos procesos de profesionalización diferentes. Respecto al primero, ya hemos compartido algunos hallazgos al estudiar el problema de la profesionalización médica a la luz de la historia de los médicos y los homeópatas sin diploma (Estrada-Orrego, Márquez-Valderrama, 2019, 2021; Márquez-Valderrama, 2015; Márquez-Valderrama, Estrada-Orrego, 2018). La investigación sobre la historia de la formación del monopolio de la medicina universitaria en Colombia reveló este nuevo objeto de estudio, la historia de la profesionalización de la odontología en el contexto de las demás profesiones de cuidados.

Según la sociología histórica, el monopolio de una profesión puede verificarse en el hecho de que sus representantes destacados dominen la jurisdicción exclusiva, es decir, que ejerzan el control en los aspectos académico, político, científico y ético (Freidson, 1978, p.84-94). En este sentido, la historia de la profesionalización ha mostrado ciertos rasgos comunes a la medicina y la odontología como profesiones autónomas. Esta historia no se puede comprender en todas sus dimensiones si se deja de lado la práctica que la medicina universitaria y gremial ha llamado tegüismo; es decir, el ejercicio laboral de médicos, homeópatas, dentistas, veterinarios, farmaceutas y comadronas sin diploma. Esta hipótesis se apoya en la presencia significativa y duradera en Colombia del “tegüismo”, afectando las profesiones odontológica y médica.

Las fuentes primarias consultadas para este artículo pertenecen todas al Archivo General de la Nación (AGN), Sección Archivos Oficiales, Fondo Ministerio de Salud, Serie Transferencias-teguas. Esta serie se compone de un total de 3.937 expedientes de solicitudes de licencias para el ejercicio de la “profesión”, clasificados en categorías, según la práctica del solicitante. Es importante hacer algunas precisiones sobre este acervo documental. Entre 1928 y 1990 se presentaron ante las autoridades del Ministerio de Instrucción Pública miles de solicitudes y de revalidaciones de licencias para el ejercicio de la medicina, la odontología y la partería. El 80% de ellas fueron presentadas por odontólogos permitidos. Esto no significa que conozcamos el número de médicos y de odontólogos permitidos del país, pues lo conservado en el AGN no representa todo lo que existió. Aun así, la cifra de odontólogos es significativa. Para el análisis, tomamos una muestra de casos de solicitudes de la primera mitad del siglo XX de hombres odontólogos y de todas las mujeres dentistas (siete) cuyas solicitudes reposan en este archivo.

El artículo está organizado en cuatro partes. La primera aborda la historia de la institucionalización de la odontología a través de la educación, la asociación y la legislación. La segunda expone el modelo del aprendizaje como vía de transmisión del oficio, a partir de varios casos de odontólogas. La tercera estudia las dificultades burocráticas que debían enfrentar los numerosos oficiantes sin diploma para solicitar licencia. La última analiza disputas locales por el monopolio del ejercicio.

## Institucionalizar la odontología

Durante la mayor parte del siglo XIX, no hubo en Colombia estudios formales de odontología y el oficio se transmitía entre familiares y amigos a través del modelo del “aprendiz”, es decir, el sistema en el cual alguien aprende al lado de otro dentista, fuese este último titulado o formado por algún odontólogo (Pelling, 2017). La formalización de la enseñanza fue lenta y las escuelas, en general, fueron efímeras. La primera de ellas, el Colegio Dental de Bogotá, se fundó en 1888 y tenía carácter privado. La formación duraba dos años y seguía el modelo de los colegios dentales norteamericanos. Durante el primer año tuvo ocho inscritos, y para 1894 ya se habían graduado 38 cirujanos dentistas, todos hombres (Otálora Cascante, 2017, p.278-280). De existencia fugaz, en ese mismo período aparecieron las primeras asociaciones de odontólogos: Asociación Dental de Bogotá (1887) y Asociación Dental de Colombia (1894) (Echeverri, 1952, p.149-150). Algunos integrantes de estas asociaciones tuvieron incidencia en la creación de los primeros programas de enseñanza de la odontología.

Para formar odontólogos fuera de la capital del país se estableció, en 1891, el Colegio Dental de Cartagena. Su rector, Constantino Pareja, se había formado en el Colegio Dental de Bogotá. Entre los primeros 11 graduados de Cartagena, cuatro llevaban el apellido Pareja, lo que permite suponer la fuerza de la transmisión familiar prolongada aun en la enseñanza instituida (Duque, López, 2002, p.346). Después de la Guerra de los Mil Días (1899-1902), el gobierno nacional decidió integrar los colegios dentales privados a instituciones universitarias públicas y otorgarles subvenciones para su funcionamiento. Así, en 1903, el Colegio Dental de Bogotá fue anexado a la Universidad Nacional de Colombia, y, en 1905, el de Cartagena a la Facultad de Medicina de la Universidad de Bolívar.

Ese mismo año, se expidió la primera reglamentación del ejercicio de la odontología en el país, que estipuló que podían ejercer los titulados del Colegio Dental de Bogotá o de “facultades extranjeras de reconocida idoneidad”, pero además quienes comprobaran “haber practicado durante dos años por lo menos en una oficina dental acreditada” (Colombia, 19 jun. 1905). Esta primera norma dejó abierta la puerta al ejercicio de los odontólogos sin diploma.

Los problemas presupuestales del programa de Cartagena llevaron a un funcionamiento intermitente y condujeron a su desaparición en 1910, lo que dejó al país con un único programa de formación de odontólogos. El mismo año, se creó la Sociedad Propagandística de Higiene Dental (Bogotá), que militaba por la divulgación de la higiene bucal entre los niños y, en general, en el pueblo colombiano (Sociedad Propagandista…, 1912, p.360).

En 1912, Antonio de Medinaceli, exalumno del Colegio Dental de Bogotá, decidió crear, en esa capital, una nueva institución de carácter privado, la Escuela Dental Nacional. Esta fue refrendada por el Ministerio de Instrucción Pública y autorizada para otorgar diplomas a los estudiantes que cursaran completo el pénsum de tres años. Ese mismo año, surgieron críticas al Colegio Dental de Bogotá por otorgar y revalidar títulos profesionales sin que se cumplieran los tiempos establecidos. Las disputas por el monopolio de la profesión se abrían paso y, desde la revista *La Odontología Colombiana,* se debatía la compleja situación de los graduados y la necesidad de luchar contra el “empirismo” y los “pseudo-dentistas” o “sacamuelas” (Otálora Cascante, 2017, p.281-285).

La ley n.83 de 1914, que reglamentó las profesiones médicas, estableció que solo podían ejercer la profesión de cirujano dentista los diplomados por la Escuela Dental Nacional o por el Colegio Dental de Bogotá. Sin embargo, gran parte de los dentistas en ejercicio no poseían diploma. De ahí que, para formalizar el oficio, esa misma ley reguló la práctica empírica mediante la exigencia de certificados: demostrar un ejercicio de más de cinco años, presentar avales de idoneidad de al menos dos cirujanos dentistas diplomados y declaraciones de buena conducta (Colombia, 23 nov. 1914). A estos dentistas se les denominará “permitidos”. Ese mismo año, se fundó en Cartagena el Instituto Politécnico Martinez Olier, institución privada que, entre otros, impartía estudios de odontología y en la cual enseñaba Constantino Pareja (Duque, López, 2002, p.377).

La enseñanza de la odontología tuvo muchos altibajos durante la década de 1920. El Colegio Dental de Medellín fue creado en 1919 por integrantes de la Sociedad Dental de Medellín (1918) y cerró en 1925 por escasez de financiación y de alumnos (Arango Botero, 1991, p.61-62). En la misma década, de acuerdo a las normas internacionales, se estableció el requisito de diploma de bachiller para el ingreso a los programas dentales, lo que llevó a un descenso sustancial de las matrículas y obligó al gobierno a revertir temporalmente esa norma. También se creó la Facultad Dental de Cartagena (1920-1938), se cerró la Escuela Dental Nacional (1924), se clausuró temporalmente el programa de odontología del Martínez Olier (1926), se liquidó el Colegio Dental de Bogotá (1927) y se estableció de forma definitiva la Facultad Dental Nacional anexa a la Facultad de Medicina de la Universidad Nacional (1927) (Duque, López, 2002, p.351-378; Otálora Cascante, 2017, p.287-289). Se establecieron sociedades dentales que buscaron divulgar y dignificar la profesión. Se destaca entre ellas la Asociación Dental de Antioquia (1923) entre cuyos asociados había dentistas permitidos (Echeverri, 1952, p.153).

A diferencia de Brasil, donde las mujeres pudieron acceder a formaciones en odontología desde 1903 (Mott et al., 2008, p.102-103), en Colombia comenzó en 1932,^1^ cuando se creó la Escuela Dental de Medellín, que permitió la inscripción de mujeres. Esto fue duramente criticado por los alumnos hombres, quienes señalaron “daños graves e inconvenientes” por compartir estudios con ellas y solicitaron que fueran separadas de las clases. Las directivas de la escuela rechazaron la solicitud y uno de sus miembros señaló que la educación mixta era habitual en los colegios dentales norteamericanos. En 1935, la escuela graduó su primera cohorte con 16 odontólogos, entre ellos cuatro mujeres (Arango Botero, 1991, p.62). Sin embargo, el cierre de la institución ese mismo año ralentizó el acceso de las mujeres a este tipo de formación.

Dado el frágil escenario institucional que graduaba pocos dentistas, gran parte de la población siguió buscando los cuidados de salud oral entre empíricos o formados por el modelo de aprendizaje. En cualquier caso, fuesen diplomados o permitidos (con licencia), el número de odontólogos era pequeño en proporción a la población. Por ejemplo, en 1934, en el departamento de Antioquia, 54 de sus 98 municipios declaraban no tener ningún oficiante de odontología; es decir, más de 350 mil habitantes no contaban con esta atención. En el cálculo nacional, la cifra ascendía a más de cuatro millones; es decir, cerca de la mitad de la población no tenía acceso a odontólogo (Colombia, 1935). En el censo de 1938, al informar la actividad económica, 1.560 hombres y 97 mujeres declararon como su fuente de ingresos el ejercicio de la odontología (Contraloría…, 1942, p.156). La precariedad de los estudios formales deja suponer que gran parte de estos declarantes eran no diplomados. Esta es solo una de las evidencias de la importancia de conocer las luchas de los permitidos por ejercer legalmente y la débil lucha de los titulados por contrarrestarlos.

Los odontólogos diplomados no solo toleraban a los permitidos, sino que los apoyaban con certificados de idoneidad que estos últimos utilizaban para solicitar o revalidar sus licencias (Colombia, 23 nov. 1914, p.83, 28 nov. 1929). Con el decreto n.2022 de 1930 se creó un organismo especializado para examinar los diplomas y las solicitudes de licencias de ejercicio de la odontología: la Junta Central de Títulos Odontológicos (JCTO), con sede en Bogotá (Colombia, 15 dic. 1930). Esto obligó a un nuevo proceso de regularización de las licencias de los odontólogos permitidos, muchos de los cuales ejercían desde hacía más de diez años. Esta nueva reglamentación muestra un avance en el control de los odontólogos sobre su propia profesión, pues los trámites ya no pasaban por una junta compuesta exclusivamente por médicos.

A partir de la ley n.51 de 1937 se estableció que los odontólogos permitidos solicitantes de licencia o renovación debían probar diez años consecutivos de ejercicio anteriores a la expedición de esa ley. Aunque antes de 1930 el “odontólogo permitido” existía de hecho, la reglamentación de esta década lo define como categoría legal y limita la jurisdicción de la expedición de licencias a la JCTO (Colombia, 15 dic. 1930, 27 jul. 1937).

Muchos odontólogos permitidos eran personas nacidas entre 1880 y 1900 que, en 1937, llevaban años de ejercicio y a quienes se les estaba exigiendo otra vez que probaran su idoneidad. A diferencia de las licencias que, con gran autonomía, en el pasado habían otorgado los gobernadores de departamentos, estas de los años 1930 eran licencias controladas por una entidad nacional. La JCTO dependía del Ministerio de Educación y recibía las solicitudes diligenciadas por las juntas seccionales departamentales, que estudiaban las solicitudes de sus respectivos municipios y transferían algunas a Bogotá, recomendadas o no. Como se verá, los trámites burocráticos eran engorrosos y, a veces, infructuosos. Con frecuencia, los dentistas se veían obligados a contratar abogados de la capital que los representaran ante la JCTO, cuya parsimonia era similar a la de su homóloga, la Junta Central de Títulos Médicos.

La institucionalización de la odontología se manifestó en este periodo en tres campos relacionados entre ellos: el de la búsqueda por establecer la enseñanza formal, el de los esfuerzos por organizar asociaciones gremiales o divulgativas y el de la regulación estatal del oficio mediante juntas de títulos profesionales.

## Un oficio aprendido y practicado en familia

El caso de Rosa C. Márquez Voinchet es ilustrativo de la odontología como oficio aprendido y practicado en familia. Era hija de Clarisa Voinchet y Lorenzo Márquez, hermano de Clodomiro. Ambos figuraban, el uno en Medellín y el otro en Sonsón, como “hábiles dentistas” (Arango, 1973, p.21). De acuerdo a una publicidad de 1906, Lorenzo ejercía como dentista desde 1869 y compartía gabinete con su hijo. La publicidad no se refería a una hija, de ahí que no se sabe si Rosa tenía un hermano dentista (Silva, 1906). Lo que parece cierto es que ella aprendió el oficio en el seno de su familia y, según su propio testimonio, lo ejercía desde 1900. Fue solo en 1928, tras la visita de un inspector de sanidad a su gabinete dental seguida del cierre del local, que se vio obligada a tramitar por primera vez una licencia. Declaró que ejercía el oficio de forma honrada, sin que nadie en todos esos años hubiera presentado queja alguna en su contra y que, además, de allí derivaba su sustento. Según ella, la sorprendió la nueva normativa en que se apoyaron para cerrar su gabinete, como medida de control del ejercicio ilegal de la odontología. También se mostró recién enterada de la existencia de la Junta Seccional de Títulos Profesionales, que concedía licencias a oficiantes sin diploma (AGN, c.1607, f.9).^2^

Para enfrentar esas medidas, Rosa le envió un memorial al gobernador del departamento de Antioquia, quien presidía la Junta de Títulos. Además de sus descargos, envió la declaración de dos cirujanos dentistas, graduados, certificando que ella practicaba la odontología desde 1900 y era “muy hábil para ejercer dicha profesión” (AGN, c.1607, f.10). En septiembre de 1928, la junta le otorgó la licencia, revalidada en procesos posteriores (1931, 1937, 1947) (AGN, c.1607, f.10). El expediente permite constatar que esta mujer, nacida en una familia de odontólogos – padre, tío, hermano –, ejerció la profesión durante casi 50 años en la ciudad de Medellín.

Otros casos de mujeres odontólogas, que aprenden el oficio en familia y solicitan licencia, los encontramos en los expedientes de Carmenza Andrade, Carlina Becerra y Carmen Rosa Arbeláez. A causa de la entrada en vigencia de la ley n.51 de 1937, Carmenza Andrade (nacida en 1910) presentó, en 1943, una solicitud de licencia ante la Junta Seccional de Títulos Odontológicos del Huila (AGN, c.111, f.4). Autoridades del municipio de Villavieja certificaron que ella y su padre, Antonio María Andrade, trabajaron juntos como odontólogos entre 1926 y 1932 (AGN, c.111, f.25). Antonio María también era odontólogo permitido y ejercía en la región huilense desde comienzos del siglo XX (AGN, c.112).

Varios declarantes del municipio donde residía Carmenza confirmaron que había ejercido la odontología desde 1927 en varios pueblos de Tolima, Caldas y Huila (AGN, c.111, f.6-9). También presentó declaraciones juramentadas de odontólogos graduados certificando su idoneidad para ejercer la odontología “sin peligro para la sociedad”, en “operatoria, prótesis, anestesia y exodoncia” (AGN, c.111, f.11-12). La odontóloga acompañó estas declaraciones de otras donde ochenta vecinos de diversos municipios certificaban su buena conducta y excelentes trabajos. A pesar de las pruebas, en noviembre de 1943, la JCTO le negó la licencia alegando que su abogado había presentado la solicitud de forma extemporánea. Pocos días después, el abogado apeló (AGN, c.111, f.13-18). El trámite de este recurso fue muy lento, y, en 1950, le fue negada de nuevo la solicitud, ya no por extemporánea, sino por carecer de pruebas certificando que había trabajado en odontología durante 10 años consecutivos (AGN, c.111, f.22).

A veces pueden parecer absurdos los obstáculos, cada vez más frecuentes e insidiosos, que ponía la JCTO, y quizás podían obedecer a presiones de los diplomados bien representados en la junta, para proteger intereses profesionales. Cada vez más, con apoyo en la ley, la junta estorba el ejercicio de los permitidos.

Frente a las dificultades, en 1951, Carmenza le otorgó poder al abogado Max Galvis, célebre por sus logros en la obtención de licencias para ejercer sin diploma la medicina, la homeopatía y la odontología. Entre los argumentos de Galvis para apelar, se destaca que el ministerio no había tenido en cuenta una sentencia del Consejo de Estado, proferida en 1931, en favor de los homeópatas, la cual señalaba que no se podía negar, intempestivamente, el derecho adquirido a ejercer la medicina ni aplicar la ley de manera retroactiva a quienes ya habían sido reconocidos legalmente en su oficio (AGN, c.111, f.24-27). Si a los homeópatas con licencia se les respetaba el derecho adquirido, por esa misma vía, se les debía respetar a los odontólogos con licencia. La apelación de Galvis fue denegada y fueron necesarias otras, hasta que, en 1954, y luego de 11 años de batallar, la Sección Jurídica del Ministerio de Salud Pública le otorgó la licencia a Carmenza Andrade (AGN, c.111, f.34).

En contraste con los trámites de Carmenza Andrade, sorprende la rapidez (menos de 2 años) con la cual Carlina Becerra de Silva logró obtener su licencia, que había solicitado casi al mismo tiempo que lo hiciera su esposo, el también odontólogo permitido, Juan N. Silva Uribe (AGN, c.263, f.2). Aunque los esposos firmaron juntos una carta al director de la JCTO, sus expedientes no muestran muchos detalles de los trámites. Llama la atención que ambos trabajaran como odontólogos permitidos y que Carlina hubiera comenzado a los 17 años. No sabemos cuándo se casaron y el expediente no revela su relación con el aprendizaje, pero es clara, en este caso, la reiteración del carácter familiar del oficio (AGN, c.263, f.11-19).

Por otra parte, el expediente de Carmen Rosa Arbeláez (nacida en 1906) pone en evidencia otra vez el aprendizaje del oficio en familia. A ello se suma que el reconocimiento del trabajo de su padre como odontólogo forma parte de los argumentos de su solicitud, una forma de trasladar el capital simbólico de él hacia ella. Según declaraciones del alcalde de Chinácota, Carmen Rosa había aprendido la profesión desde 1926, junto a su padre, el dentista José María Arbeláez, con quien estuvo practicando, hasta que él falleció y ella decidió ocuparse del gabinete sola. Puesto que en ese momento en su localidad no estaba ejerciendo odontólogo titulado y que, durante 12 años, ella había atendido a su clientela sin que se presentaran contingencias, el alcalde respaldó su solicitud, subrayando que ella era “un elemento distinguido de la ciudad”, apreciada por sus méritos y virtudes (AGN, c.123, f.12). Su solicitud también fue respaldada por el párroco, por dos vecinos que certificaron su conducta intachable y dos odontólogos graduados de otros municipios que declararon que ella llevaba 26 años ejerciendo la profesión (AGN, c.123, f.7-12).

Carmen Rosa comenzó el proceso de solicitud de licencia en marzo de 1952 y en noviembre le otorgó un poder a un abogado para que la representara en Bogotá ante la JCTO. En julio de 1954 se emitió una constancia de que la solicitud estaba en proceso, la licencia solo le fue concedida en enero de 1957 (AGN, c.123, f.15-22).

Si el número de solicitudes de mujeres se compara con el de solicitudes de hombres, es evidente la escasez de las primeras en el archivo. Seguramente esta exigüidad tiene relación con que en la primera mitad del siglo XX el espacio propio de lo femenino era el hogar y sus actividades debían limitarse al ámbito doméstico, de ahí que los pocos expedientes de mujeres cobren aún más valor como testimonio de la participación y de los esfuerzos de ellas por ejercer oficios y profesiones reservadas a los hombres. Además, revelan que la formación de un oficio afín a la medicina por la vía maestro-aprendiz también les concierne.

## La maraña burocrática

Aunque no sabemos si son hijos de un odontólogo y poco o nada se conoce en Colombia acerca del origen de su apellido francés, los tres hermanos Thevenin, nacidos en el mismo lugar, en zona rural interna de la región atlántica, practicaron la odontología en tres localidades vecinas a comienzos del siglo XX. Dejaron huella en el archivo a causa de la entrada en vigencia de la ley n.51 de 1937 y son un ejemplo más de la tortuosa y flemática maraña burocrática.

El mayor de ellos, Ernesto Thevenin Gracia, nacido en 1899, solicitó revalidación de su licencia, en 1938, y para ello otorgó poder en Cartagena a un abogado (AGN, c.2825, f.1-2). Presentó los documentos exigidos por la ley. Probó su ejercicio de más de 10 años consecutivos; su idoneidad fue certificada por dos odontólogos diplomados, y varios vecinos notables atestiguan su honorabilidad (AGN, c.2825, f.7-9).

Sin avanzar en los trámites, la JCTO le retuvo la documentación aduciendo que su apoderado no había comprobado la fecha de presentación de la solicitud ante la Junta Departamental de Títulos de Bolívar; tampoco había justificado el registro oficial en el Ministerio de Educación Nacional (MEN) de los diplomas de los odontólogos que certificaron su idoneidad (AGN, c.2825, f.12). Esto último suponía un procedimiento adicional para quienes tramitaban la licencia (Colombia, 1937). Una solicitud presentada ante la JCTO, en junio de 1941, muestra la lentitud de la diligencia y su reactivación en ese año. Pero en marzo de 1944, las diligencias seguían estancadas, pues Ernesto otorgó poder a otro abogado residente en Bogotá para continuar las gestiones. En ese mismo mes, la JCTO le concedió licencia (AGN, c.2825, f.14-16).

El segundo de los hermanos, Aníbal, nacido en 1900, ejercía la dentistería desde 1925 en Cereté y desde 1927 en Montería, como lo declararon en 1938 dos “comerciantes” y un “farmaceuta”, “vecinos notables” de ese lugar que destacaron los 10 de años de ejercicio “sin interrupción y con gabinete propio” (AGN, c.2822, f.1-4). Aníbal también se vio obligado a tramitar la renovación de su licencia en 1938. No podemos precisar el resultado de la gestión, pero sabemos que en 1949 inició otra demanda. Y en esta relata haber perdido los documentos de su anterior solicitud, debido a la destrucción de las oficinas públicas durante “el Bogotazo” (9 de abril de 1948) (AGN, c.2822, f.19). Esta última solicitud le fue negada. Sin embargo, en mayo de 1954 la resolución de rechazo fue anulada, y el Ministerio de Salud Pública le otorgó licencia mediante la tercera gestión de un tercer abogado.

El menor de los tres hermanos, Daniel, nació en 1907. Ejercía la odontología en Sahagún. En agosto de 1945, el cirujano dentista Edmundo Pizarro, rector de la Facultad Dental de Cartagena, certificó que Daniel era “competente y técnico en operatoria, prótesis dental, exodoncia y curso anestésico” y que ejercía el oficio desde 1927 (AGN, c.2823, f.1-5). En 1946 el alcalde de Sahagún certificó que Daniel había ejercido la odontología durante 19 años en ese municipio (AGN, c.2823, f.4). El año siguiente, el rector de la Universidad de Cartagena, Francisco Obregón Jarava, declaró que conocía desde hacía varios años a Daniel Arturo Thevenin y que “siempre” le había visto “ejercer con notoria competencia la profesión de odontólogo” (AGN, c.2823, f.6). Los demás folios del expediente de Daniel muestran que en 1954 los trámites continuaban (AGN, c.2823, f.7-20).

A finales de 1937, para no trabajar en la ilegalidad, numerosos odontólogos sin diploma se vieron obligados a certificar “más de 10 años” de ejercicio contados desde ese momento hacia atrás. En muchas de las solicitudes de licencia se hace evidente un obstáculo interpuesto por las autoridades de Bogotá, el del plazo dentro del cual debía presentarse la solicitud. La JCTO provocó a partir de ahí una confusión y durante años solo aceptó tramitar las solicitudes presentadas antes de la promulgación del decreto n.32 de 1938 (reglamentario de la ley n.51 de 1937). El caso del odontólogo permitido Miguel A. Toro fue determinante, pues con él la exigencia de alguna fecha a los solicitantes fue demandada ante el Consejo de Estado como un atentado al derecho individual al trabajo. En una sentencia del 20 de marzo de 1952, ese tribunal falló a favor de los odontólogos permitidos, estableciendo que todos los ciudadanos que cumplieran los requisitos de ley tenían el derecho a continuar ejerciendo la odontología, mediante la presentación, “en cualquier tiempo” de los documentos probatorios. A mediados de 1954, el apoderado de Daniel Thevenin concluyó en su memorial que la exigencia de su representado era un derecho, que la fecha de la solicitud era inocua y no existía razón alguna para limitar las peticiones fundamentadas en esa ley (AGN, c.2823, f.21).

Los trámites de las licencias de los dos hermanos mayores tardaron 6 años en cada caso. Al menor le tomó 9 años obtenerla. Los tres tuvieron que apoderar abogados que los representaran ante la parsimoniosa JCTO, lo que significó un aumento en los plazos y en los costos de los trámites. Además, ser representado por un abogado no garantizaba el éxito, como se constata en el caso de Aníbal, quien tuvo que contratar sucesivamente a tres diferentes. Los tres hermanos padecieron la maraña burocrática para trabajar legalmente en poblaciones vecinas a su lugar de nacimiento, y es probable que los débiles controles estatales no les hayan impedido ejercer durante los prolongados intervalos de las gestiones administrativas que los dejaban en la ilegalidad.

Hay que subrayar que, en Colombia, la presencia prolongada de los odontólogos permitidos obedece en parte a la ausencia de atenciones y cuidados en un territorio muy extenso de geografía abrupta, deficientes vías de comunicación y con una población mayoritariamente rural. De ahí que en muchos de los casos las licencias se aprobaran por ausencia de odontólogos titulados en los municipios donde ejercían los permitidos. Entre estos había personas de escasa instrucción a quienes se les dificultaron aún más los trámites.

Cuando las personas del pueblo se dirigían a las autoridades, las distancias no eran sólo geográficas, sino también culturales. Es el caso de Flora Aragón, quien, a finales de 1950, intentó tramitar una licencia para el ejercicio de la “técnica de fabricación de cajas de dentistería” que, según ella, llevaba 22 años practicando en Dagua (Valle del Cauca). La distancia cultural se nota en la defectuosa redacción de sus comunicaciones, exceso de cortesía y adulación, con las cuales se dirige al ministro de Higiene, y no a la JCTO. Este escaso capital simbólico se hace evidente en su exigua solicitud, que contiene argumentos inusuales: declara ser “mujer casada madre de muchos hijos”, todos los cuales prestaron servicio militar y, además, que en Dagua no hay nadie más que practique la dentistería. Cuando Flora habla de su oficio se refiere solo a la mecánica dental o fabricación de prótesis. En cambio, cuando algunos vecinos de su pueblo testifican a su favor, se refieren a ella como dentista. Cumple ciertas condiciones, como ser la única dentista y tener aceptación en su pueblo. Sin embargo, en 1952, la JCTO le responde que no tramitará su incompleta solicitud (AGN, c.80).

El caso de Gilma Velásquez, quien trabajaba como dentista en Tocaima y Sesquilé (Cundinamarca), es semejante porque inicia el trámite con un expediente incompleto y con unas certificaciones insuficientes, pues solo logra probar 3 años de ejercicio. La respuesta del Consejo Nacional de Práctica Profesional fue no adelantar ningún trámite (AGN, c.3038).

Contrastan estos dos expedientes de mujeres dentistas con el de Kotrina Pikcilingis, mujer rusa-lituana que a los 34 años de edad presentó solicitud completa para ejercer como odontóloga extranjera en Colombia. Adjuntó su diploma original de cirujano dentista de una universidad lituana, la traducción oficial de este, las calificaciones y la refrendación de este por la Organización Internacional para los Refugiados adscrita a la Organización de las Naciones Unidas. Kotrina llegó en 1949 a Colombia con otros veinte lituanos huyendo de la invasión rusa. El abogado que la representa solicitó un trato excepcional favorable para “las víctimas del comunismo” (AGN, c.225, f.1). El Ministerio de Relaciones Exteriores colombiano certificó la autenticidad de los diplomas. Las posibilidades de ejercicio de Kotrina en Colombia se limitaban, en sus 3 primeros años de residencia, al trabajo como odontóloga en zonas rurales, pues el decreto n.3.842 de 1949 establecía esa condición para todo inmigrante proveniente de un país sin convenio de canje de títulos universitarios con Colombia. Kotrina inició el trámite de su licencia a comienzos de 1949 y sólo la obtuvo en octubre de 1952 (AGN, c.225, f.1).

La rigidez de los trámites es una manifestación del poder obstaculizador de una junta compuesta por profesionales que defendían, desde la capital, una jurisdicción monopólica sobre el ejercicio de la odontología. A pesar de estos obstáculos burocráticos, en lo jurídico, específicamente en la protección del derecho al trabajo, los permitidos a veces ganaban la batalla, mientras que los prolongados plazos para obtener la licencia dejan pensar que ejercían sin ella. Restringir la jurisdicción era muy difícil en la práctica, porque ¿cómo controlar todo el tiempo la vida de tantos pueblos lejanos?

## Odontólogos diplomados defienden su monopolio

Uno de los factores que movía a los odontólogos permitidos a tramitar sus licencias y a los diplomados a refrendar sus diplomas era la presión que ejercían los titulados agremiados ante la JCTO. Considerando que la serie documental analizada conserva sobre todo expedientes de odontólogos y médicos permitidos, el hallazgo de algunos de odontólogos diplomados da indicios de: (1) las tensiones entre estos y la validación de la enseñanza formal; (2) el control estatal del ejercicio de la odontología y (3) la considerable presencia de los permitidos. Como hemos mostrado, estos últimos se enfrentaban a largos y complicados trámites, pero también a la oposición de los diplomados que buscaban obtener el monopolio del ejercicio y que además estaban obligados por la ley a refrendar sus títulos de cirujano dentista después de graduarse.

El expediente de Manuel Almonacid, odontólogo titulado que ejercía en la Intendencia de San Andrés y Providencia (AGN, c.18), territorio insular muy alejado de la capital, aporta un caso revelador. Primero, algunos problemas relacionados con la validación de un diploma de “cirujano dentista” expedido en Colombia; segundo, la lucha por hacer respetar como derecho un monopolio de ejercicio en el archipiélago; por último, el carácter incipiente de la formalización de los estudios de odontología en el país.

Las primeras vicisitudes de Almonacid están relacionadas con errores cometidos por la administración nacional en la elaboración del decreto n.1.759 de 1931, norma que legitimaba los títulos de cirujano dentista otorgados por el “Instituto Martínez Olier” y por la “Facultad Dental de Cartagena”, entre noviembre de 1923 y noviembre de 1927. Sin embargo, en el texto los nombres de ambas instituciones aparecen cambiados. Así, cuando Almonacid procedió a refrendar su diploma, esos errores oficiales fueron un obstáculo para el reconocimiento de su título.

En una carta de mayo de 1932, dirigida al ministro de Educación, Almonacid quiso aclarar el problema de su diploma, expedido en junio de 1927 por la Facultad Dental de Cartagena. Según Almonacid, el Ministerio de Instrucción Pública, desde 1921, “reconoció oficialmente los diplomas que para el ejercicio de la odontología expidiera” esa facultad (AGN, c.18, f.2). También citó el artículo primero del mencionado decreto de 1931, texto que contenía los errores de escritura. Cuenta Almonacid que en 1929 “la Facultad Dental de Cartagena cesó de funcionar” y que él no pudo hallar a las personas que en aquel entonces fungían como rector y secretario, quienes habían firmado legalmente su diploma (AGN, c.18, f.3). El director de educación pública del departamento de Bolívar certificó que en Cartagena sí funcionó (de 1920 a 1929) la “Facultad Dental de Cartagena”; que allí nunca funcionó algo llamado “Escuela Dental de Cartagena”; acreditó la autenticidad de las firmas y del diploma de Almonacid (AGN, c.18, f.4). Con estas diligencias ante autoridades departamentales y nacionales, en 1932, Almonacid obtuvo el reconocimiento de su diploma y pudo registrarlo en el MEN. Más allá de lo fáctico, estos eventos muestran la inestabilidad institucional característica de los primeros años de la formalización de los estudios de odontología en Colombia.

En su calidad de odontólogo diplomado e integrante de la Junta de Títulos de la Intendencia de San Andrés, en abril de 1947, Almonacid inició gestiones para impedir que un dentista permitido continuara ejerciendo en el corregimiento Isla de Providencia (AGN, c.18, f.8). En marzo de 1948, Almonacid insistió con diligencias ante el MEN y expresó en un memorial: “Tengo conocimiento de que ha llegado a ese lugar, a establecerse en él, el tegua Roudolph Newball Jr. ..., como profesional de Dentistería, y que al efecto ya empezó a ofrecer sus servicios profesionales a algunos de mis clientes” (AGN, c.18, f.9). En otro memorial dice: “Este profesional se ocupaba no hace mucho tiempo en la fabricación de escobas y ahora obtenta [*sic*] título de odontología, siendo manifiesta su impreparación y por otra parte carece de título que justifiquen [*sic*] su idoneidad” (AGN, c.18, f.7).

Seguro de su título y celoso de proteger su profesión, exigió en sus comunicaciones que se le impidiera el ejercicio de la odontología a Newball, en cumplimiento de las disposiciones legales. Las diligencias generadas por esta denuncia siguieron su curso, y el 13 de febrero de 1948 el tegua Newball declaró ante el juez de San Andrés que no poseía título de odontólogo. Le impusieron la multa correspondiente y le prohibieron ejercer el oficio de dentista. Sin embargo, un mes después, la JCTO se mostró favorable para que el tegua Newball siguiera ejerciendo en Providencia, por cuanto allí no había quien realizara ese tipo de trabajo y la comunidad lo necesitaba. En efecto, en marzo de 1948, el secretario general del Ministerio de Higiene de Colombia, Roberto Serpa, ordenó mediante radiograma revocar la disposición que prohibía a Newball ejercer, mientras no hubiera odontólogo graduado en la Isla de Providencia (AGN, c.18, f.9-11).

La Junta Seccional de Títulos Odontológicos de San Andrés y Providencia, a la cual pertenecía Almonacid, reunida en pleno, se acogió a la orden de Bogotá y no se sabe si la querella continuó (AGN, c.18, f.12). Como en numerosos casos, en este se puede concluir que la ausencia de profesional graduado en una localidad era un motivo de peso para legitimar el trabajo de un tegua.

También de San Andrés, hay otro expediente de cirujano dentista titulado, el de Bedel Duffis (AGN, c.740, f.3), quien, en 1936, se puso en evidencia ante las autoridades por un supuesto error de procedimiento clínico, que tuvo que ser subsanado por otro profesional. Este expediente contiene uno de los pocos testimonios del sufrimiento de una paciente a causa de una dolencia bucal. A la señora de 26 años de edad, Duffis le practicó una exodoncia, aparentemente, mal hecha, que le dejó restos de raíces de un molar en la encía. El intenso dolor movió a la dama a consultar a un segundo odontólogo el mismo día en que Duffis la operó. Adora Reed consultó a Almonacid, quien, después de examinarla, le dijo que no podía extraerle esas raíces por estar muy profundas. Doce días después, en vista de que el dolor no la dejaba descansar, la señora Reed volvió a consultar a Almonacid y este dentista “le aplicó anestésico y le extrajo dos raíces correspondientes a una cordal inferior derecha” (AGN, c.740, f.4). Más allá de lo anecdótico, esta doble consulta hizo visible la rivalidad entre dos cirujanos dentistas diplomados y ejerciendo en la misma localidad. Manuel Almonacid, en su calidad de integrante de la Junta de Títulos, aprovechó la ocasión de recibir en su clínica dental a una paciente sufriente para denunciar la supuesta incompetencia de su rival Bedel Duffis.

En los dos municipios que constituyen esta intendencia, es decir los de San Andrés y Santa Isabel de Providencia, ejercen la profesión de dentista varias personas que no tienen diploma ni reúnen los requisitos exigidos por el inciso a) del artículo 1º de la ley 51 de 1937, ni los exigidos por los decretos números 361 y 453 de 1933. … Ejerce la odontología general en San Andrés el señor Bedel Duffis y practican abusiones dentales con y sin anestésico en Santa Isabel de Providencia, los señores Víctor R. Howard y Clinton Bryan, y la señora Anna H. de Archbold (AGN, c.740, f.7).

Llama la atención que tras denunciar a cuatro dentistas (según él ilegales) el mismo Almonacid explica que solamente adjuntó pruebas del ejercicio ilegal de la odontología de Bedel Duffis (AGN, c.740, f.7). Este raizal denunció ante la JCTO que en la Junta Seccional de Títulos de San Andrés y Providencia había “dos profesionales” interesados en “hacer desconocer” los títulos de cirujano dentista legal de “los individuos naturales y establecidos”. Declaró, además, que, entre 1929 y 1936, la Junta de Títulos de San Andrés solo se reunió una vez y durante ese periodo nunca se interesó en la verificación de la legalidad de su diploma ni en su idoneidad. Cuenta que trabajó en “cirugía dental” bajo la tutela de César Neyra Borda, odontólogo graduado de la “Facultad Nacional” de Colombia y de una universidad norteamericana, pero que dejó de practicar la profesión cuando el doctor Neyra se retiró del archipiélago (AGN, c.740, f.11).

Duffis había estudiado en el Colegio Dental de Medellín y afirmó que su rector, el doctor Abel Uribe Jaramillo, podía atestiguar sobre su “aprovechamiento científico”. Afirmó además que el dentista Manuel Almonacid estaba interesado en hacerle perder su “derecho de ejercer la profesión” y que fue eso lo que lo motivó a solicitar una refrendación de su título de cirujano dentista expedido en Medellín el 1º de julio de 1923 (AGN, c.740, f.8, 11).

Aunque fragmentario de este expediente se infiere que Duffis pudo probar que era cirujano dentista diplomado, pero no indica qué resolvió la JCTO frente a las acusaciones de Almonacid contra Duffis ni frente a la solicitud de este último. Los conflictos de jurisdicción no solo se expresan en el funcionamiento centralizado de la JCTO, sino que se vivían también en la cotidianidad de las regiones apartadas. Los diplomados en ejercicio querían hacer valer su monopolio en contra de permitidos y de otros titulados.

## Consideraciones finales

Para los odontólogos en Colombia, ejercer el control de la profesión significó definir, legalmente y en la práctica, quién decidiría sobre el derecho de ejercicio. Aunque la tomaran como modelo, debieron enfrentarse a la hegemonía académica, política, científica y ética de la profesión médica y liberarse de su tutela. Para ello, debieron apropiarse de tres facetas institucionales que les permitieron debatir sobre el estatuto de su profesión: la asociación en torno a una identidad de gremio, la formalización de estudios especializados y la representación activa en las juntas de títulos.

En la iniciativa de conformar asociaciones antecedieron a los médicos (1887 y 1894). Sin embargo, las primeras sociedades de odontólogos no tuvieron como objetivo principal la lucha contra los empíricos, sino que se enfocaron en crear los primeros estudios formales, divulgar ampliamente la higiene bucal y dignificar la odontología como profesión. Una singularidad del caso colombiano es que los odontólogos diplomados fueron muy permisivos con los que ejercían sin diploma, algunos de estos incluso formaron parte de las primeras asociaciones de odontólogos.

En cuanto a instituir la enseñanza especializada, los odontólogos iban a la zaga de la medicina, que había creado y puesto a funcionar varias facultades en el siglo XIX. Ya señalamos los escollos en la constitución de escuelas de odontología estables y duraderas que permitieran un ritmo de reproducción de los conocimientos con relevo generacional. Aunque hubo intentos de normalizar la profesión mediante la educación formal, al mismo tiempo, el Estado promulgaba normas laxas frente a los empíricos. Esto repercutió en la actividad gremial y en el sistema de enseñanza, ambos débiles, pues ayudó a mantener la connivencia prolongada, sin exclusividad cognitiva, entre los practicantes de todo tipo, impidiendo que las competencias científicas del odontólogo diplomado se perfilaran con nitidez.

Las leyes que regulaban el ejercicio de la odontología mantuvieron en sus textos, por muchos años (1905-1954), un estatuto de odontólogos con “licencia” y sin diploma, demarcado por la categoría “odontólogo permitido”. Haber establecido esta categoría sugiere la existencia de varios tipos de odontólogos o al menos obliga a diferenciar a los titulados entre otros practicantes. La prolongada convivencia entre diplomados y permitidos explica, en parte, la vaguedad de los límites de acción de estos últimos, lo cual constituye una peculiaridad del mercado profesional de la dentistería en Colombia. Es probable que esto estuviera relacionado con la escasez de odontólogos titulados en el territorio nacional y con el carácter artesanal, comercial y familiar del oficio.

Los permitidos eran numerosos, mientras que los titulados escaseaban. Ante esta realidad, la JCTO, creada en 1930, se convirtió en un apretado filtro del proceso de obtención de licencias. El centralismo obstaculizó e hizo aún más lento e inoperante el sistema de control de títulos y licencias: entorpecía los trámites, lo que podía proteger la jurisdicción de los titulados; pero, paradójicamente, también dejaba enormes zonas grises donde podían operar los irregulares.

En esa misma década, se permitió el ingreso de mujeres a una de las escuelas de odontología, lo que generó resistencias de los alumnos hombres. Sin embargo, el proceso de incorporación de las mujeres a la odontología queda por estudiar. Los pocos indicios nos muestran que fue tímido, lento y lleno de obstáculos, pues la primera escuela que se atrevió a desafiar la resistencia masculina fue muy efímera. Por otra parte, según el archivo y la estadística oficial de ocupaciones, parece que el modelo de aprendizaje hubiera sido más efectivo que la universidad en cuanto a hacer ingresar mujeres al oficio, pues los pocos expedientes hallados (siete) muestran a cuatro de ellas como personas que aprendieron y practicaron la odontología bajo la tutela de hombres, casi siempre de su misma familia.

## NOTAS

1 En 1930, desde la Facultad de Odontología de la Universidad Nacional, se propuso la creación de una Escuela de Higienistas Dentales para el “entrenamiento científico de señoritas” que trabajarían como *nurses* y “secretarias” en servicios dentales de escuelas públicas y privadas, establecimientos de beneficencia, sociedades industriales y clínicas dentales particulares (Duque, López, 2002, p.414). Aunque no se concretó, la propuesta revela la intención de destinar para mujeres oficios de salud, subordinados al trabajo masculino.

2 De aquí en adelante, se citará la serie Transferencias-teguas, así: AGN, c. indicando el número de la carpeta, y f., el número de folio.

## References

[B1] AGN, Archivo General de la Nación (Colombia). Sección República, Fondo Ministerio de Salud, Serie teguas. Carpetas: 18, 80, 111, 112, 123, 225, 263, 740, 1607, 2822, 2823, 2825, 3038 (Archivo General de la Nación, Bogotá). 1928-1990.

[B2] AGOSTONI, Claudia. Médicos científicos y médicos ilícitos en la ciudad de México durante el Porfiriato. *Estudios de Historia Moderna y Contemporánea de México*, v.19, n.19, p.13-31, 1999.

[B3] ALLEVI, José Ignacio; CARBONETTI, Adrián. De vecinos sufrientes a ciudadanos peticionantes: actores e instituciones en la construcción de un arte de curar moderno en la provincia de Santa Fe (Argentina, 1847-1907). Trashumante: Revista Americana de Historia Social, n.17, p.80-103, 2021.

[B4] ARANGO, Gabriel. Genealogías de Antioquia y Caldas. Medellín: Bedout, 1973. 2v.

[B5] ARANGO BOTERO, Alberto. La escolarización de la odontología en Antioquia: bosquejo histórico. Revista Facultad de Odontología Universidad de Antioquia, v.2, n.2, p.61-69, 1991.

[B6] ARMUS, Diego. Entre médicos y curanderos: cultura, historia y enfermedad en la América Latina moderna. Buenos Aires: Editorial Norma, 2006.

[B7] ARMUS, Diego; GÓMEZ, Pablo (org.). The gray zones of medicine: healers and history in Latin America. Pittsburgh: University of Pittsburgh Press, 2021.

[B8] BELMARTINO, Susana. Historias de la profesión médica: Argentina y Estados Unidos en el siglo XX. Salud Colectiva, v.6, n.3, p.329-353, 2010.

[B9] CARRILLO, Ana María. Profesiones sanitarias y lucha de poderes en el México del siglo XIX. Asclepio, v.50, n.2, p.149-168, 1998.

[B10] CARVALHO, Cristiana Leite. A transformação no mercado de serviços odontológicos e as disputas pelo monopólio da prática odontológica no século XIX. História, Ciências, Saúde – Manguinhos, v.13, n.1, p.55-76, 2006. 10.1590/s0104-59702006000100004 17580429

[B11] COLOMBIA. Ley n.51, de 11 de junio de 1937. Por la cual se reglamenta el ejercicio de la odontología. Diario Oficial, 27 jul. 1937.

[B12] COLOMBIA. Departamento de la Contraloría. Anuario general de estadística (1934). Bogotá: Imprenta Nacional, 1935.

[B13] COLOMBIA. Decreto n.2.022, de 29 de noviembre de 1930. Por la cual se reglamenta la ley 35 de 1929 sobre el ejercicio de la odontología en Colombia. Diario Oficial, 15 dic. 1930.

[B14] COLOMBIA. Ley n.35, de 22 de noviembre de 1929. Por la cual se reglamenta el ejercicio de la profesión de medicina en Colombia. Diario Oficial, 28 nov. 1929.

[B15] COLOMBIA. Ley n.83, de 19 noviembre de 1914. Por el cual se reglamenta el ejercicio de las profesiones médicas. *Diario Oficial*, 23 nov. 1914.

[B16] COLOMBIA. Decreto 592, de 8 de junio de 1905. Por el cual se reglamenta el ejercicio de la profesión de medicina. *Diario Oficial*, 19 jun. 1905.

[B17] CONTRALORÍA General de la República. Dirección Nacional de Estadística. Censo general de población, 5 de julio de 1938: ordenado por la ley 67 de 1917. Bogotá: Imprenta Nacional, 1942. 16v.

[B18] CORREA, María José; ZÁRATE, María Soledad. Historizar la profesionalización sanitaria: perspectivas desde Chile y Argentina. Dynamis, v.37, n.2, p.263-272, 2017.

[B19] DUQUE, Camilo, LÓPEZ, Héctor. La odontología en Colombia: historia, cultura y sociedad. Bogotá: Universidad El Bosque, 2002.

[B20] ECHEVERRI, Aquiles. Historia y legislación de la odontología en Colombia. Buenos Aires: Quetzal, 1952.

[B21] ESTRADA-ORREGO, Victoria; MÁRQUEZ-VALDERRAMA, Jorge. Recognition without a diploma: the wanderings of the healer Indio Rondín in early twentieth-century Colombia. In: Armus, Diego; Gómez, Pablo (ed.). The gray zones of medicine: healers and history in Latin America. Pittsburgh: University of Pittsburgh Press, 2021. p.123-137.

[B22] ESTRADA-ORREGO, Victoria; MÁRQUEZ-VALDERRAMA, Jorge. Defensa de los derechos adquiridos: luchas y albures del ejercicio de la homeopatía en Colombia (1905-1950). História, Ciências, Saúde – Manguinhos, v.26, n.4, p.1355-1372, 2019. 10.1590/S0104-59702019000400019 31800846

[B23] FREIDSON, Eliot. La profesión médica. Barcelona: Península, 1978.

[B24] LIMA, Marcelino Carmo de; NASCIMENTO, Sulenir Cândida da Silva; ALVES, Jerônimo. Disputas pelo monopólio da prática odontológica e a criação da Escola Livre de Odontologia do Pará (1911-1914). Amazônia: Revista de Educação em Ciências e Matemáticas, v.13, n.25, p.85-99, 2016.

[B25] MÁRQUEZ-VALDERRAMA, Jorge. El médico de oficio en Colombia en las décadas de 1920 y 1930. Revista Mundos do Trabalho, v.7, n.13, p.85-104, 2015.

[B26] MÁRQUEZ-VALDERRAMA, Jorge; ESTRADA-ORREGO, Victoria. Culebrero, tegua, farmaceuta y dentista: el Indio Rondín y la profesionalización médica en Colombia (1912-1934). Anuario Colombiano de Historia Social y de la Cultura, v.45, n.1, p.79-104, 2018.

[B27] MARTIN, Ana Laura. Partear y cuidar en Buenos Aires (1877-1920): una aproximación comparativa. Anuario del Instituto de Historia Argentina, v.18, n.1, e061, 2018.

[B28] MOTT, María Lucía et al. Moças e senhoras dentistas: formação, titulação e mercado de trabalho nas primeiras décadas da República. História, Ciências, Saúde – Manguinhos, v.15, supl., p.97-116, 2008. 10.1590/s0104-59702008000500005 19397031

[B29] OTÁLORA CASCANTE, Andrés Ricardo. La institucionalización de la odontología (1888-1942). In: Restrepo-Zea, Estela (ed.). Ciencias de la vida: colección del sesquicentenario. Bogotá: Universidad Nacional de Colombia, 2017. v.1, p.276-300.

[B30] PELLING, Margaret. Managing uncertainty and privatising apprenticeship: status and relationships in English medicine (1500-1900). Social History of Medicine, v.32, n.1, p.34-56, 2017.

[B31] QUEVEDO, Emilio et al. *Historia de la medicina en Colombia*: hacia una profesión liberal (1865-1918). Bogotá: Editorial Norma, 2010. 3v.

[B32] RAMACCIOTTI, Karina. La profesionalización del cuidado sanitario: la enfermería en la historia argentina. Trabajos y Comunicaciones, n.49, e081, 2019.

[B33] SCHAPIRA, Marta V. Escenarios históricos, práctica profesional y poder: el caso de la odontología. Cuadernos de Antropología Social, n.17, p.101-115, 2003a.

[B34] SCHAPIRA, Marta V. La odontología en Argentina: historia de una profesión subordinada. História, Ciências, Saúde – Manguinhos, v.10, n.3, p.955-977, 2003b. 10.1590/s0104-59702003000300008 14994717

[B35] SILVA, Isidoro. Primer directorio general de la ciudad de Medellín para el año de 1906. Medellín: [s.n.], 1906.

[B36] SOCIEDAD PROPAGANDISTA de Higiene Dental. Estatutos de la Sociedad Propagandista de Higiene Dental. Revista Odontológica, v.2, n.9, p.360-366, 1912.

[B37] WARMLING, Cristine Maria; MARZOLA, Norma Regina; BOTAZZO, Carlos. Da autonomia da boca: práticas curriculares e identidade profissional na emergência do ensino brasileiro da odontologia. História, Ciências, Saúde – Manguinhos, v.19, n.1, p.181-195, 2012. 10.1590/s0104-59702012000100010 22488381

[B38] ZÁRATE, María Soledad. De partera a matrona: hacia la asistencia profesional del parto en Chile en el siglo XIX. Calidad en la Educación, n.27, p.284-297, 2007.

